# MicroRNA-126 Priming Enhances Functions of Endothelial Progenitor Cells under Physiological and Hypoxic Conditions and Their Therapeutic Efficacy in Cerebral Ischemic Damage

**DOI:** 10.1155/2018/2912347

**Published:** 2018-04-11

**Authors:** Qunwen Pan, Jieyi Zheng, Donghui Du, Xiaorong Liao, Chunlian Ma, Yi Yang, Yanyu Chen, Wangtao Zhong, Xiaotang Ma

**Affiliations:** ^1^Guangdong Key Laboratory of Age-Related Cardiac and Cerebral Diseases, Institute of Neurology, Affiliated Hospital of Guangdong Medical University, Zhanjiang 524001, China; ^2^College of Health Science, Wuhan Sports University, Wuhan 430079, China

## Abstract

Endothelial progenitor cells (EPCs) have shown the potential for treating ischemic stroke (IS), while microRNA-126 (miR-126) is reported to have beneficial effects on endothelial function and angiogenesis. In this study, we investigated the effects of miR-126 overexpression on EPCs and explore the efficacy of miR-126-primed EPCs (EPC^miR-126^) in treating IS. The effects of miR-126 overexpression on EPC proliferation, migratory, tube formation capacity, reactive oxygen species (ROS) production, and nitric oxide (NO) generation were determined. In *in vivo* study, the effects of EPC^miR-126^ on the cerebral blood flow (CBF), neurological deficit score (NDS), infarct volume, cerebral microvascular density (cMVD), and angiogenesis were determined. Moreover, the levels of circulating EPCs (cEPCs) and their contained miR-126 were measured. We found (1) miR-126 overexpression promoted the proliferation, migration, and tube formation abilities of EPCs; decreased ROS; and increased NO production of EPCs via activation of PI3K/Akt/eNOS pathway; (2) EPC^miR-126^ was more effective than EPCs in attenuating infarct volume and NDS and enhancing cMVD, CBF, and angiogenesis; and (3) infusion of EPC^miR-126^ increased the number and the level of miR-126 in cEPCs. Our data indicate that miR-126 overexpression enhanced the function of EPCs *in vitro* and *in vivo*.

## 1. Introduction

Cerebral ischemic stroke is one of the leading causes of death and disability in the world. Endothelial repair and neovascularization are important for functional recovery from ischemic stroke [1–4]. Endothelial progenitor cells (EPCs) could differentiate into mature vascular endothelial cells (ECs), playing important roles in maintaining vascular homeostasis and promoting angiogenesis [3]. Under vascular ischemic injury, EPCs mobilize from the bone marrow (BM) to injury area, participating in the neovascularization [1]. Reduced number and impaired function of circulating EPCs (cEPCs) are reported to be correlated to worse clinical outcome in patients with ischemic stroke [5]. EPC transfusion has shown potential therapeutic effects on ischemic stroke [6]. However, under such pathological environment, the function of transfused EPCs is usually impaired by existing risk factors [6–8]. Therefore, exploring approaches to combine autologous cell therapy with ex vivo genetic manipulations to maintain/improve their function is critical for ischemic stroke therapy.

MicroRNAs (miRs), highly conserved, single-stranded 18–25 nucleotide noncoding RNAs, could regulate target gene expression by degrading the messenger RNAs (mRNAs) and inhibit the translation of protein [9]. miR-126, highly expressed in ECs and EPCs, is a multifunctional miR playing important roles in regulating vascular integrity and promoting angiogenesis. Several studies have demonstrated that miR-126 could enhance the proliferation and migration abilities of ECs [10, 11]. However, circulating miR-126 has been shown to be reduced in vascular diseases including stroke and coronary artery disease [12, 13]. Moreover, miR-126 was downregulated in EPCs under hypoxia condition [14]. Previous study has demonstrated that knockdown of miR-126 expression impairs ischemia-induced vessel reparation [15]. Thus, we hypothesize that overexpression of miR-126 could rescue/promote functions of EPCs under hypoxic condition and enhance the therapeutic efficacy of EPCs in ischemic stroke. Meanwhile, miR-126 has been reported to regulate angiogenic process and EC/EPC function via phosphorylated phosphatidylinositol-3-kinase (PI3K)/Akt/endothelial nitric oxide synthase (eNOS) pathway [16, 17]. Downregulation of miR-126 in EPCs impairs their function through PI3K/Akt/eNOS signal pathway [18]. Therefore, we aimed to study the inner mechanism of miR-126 overexpression in the modulation of EPC survival and functions by regulating the PI3K/Akt/eNOS pathway.

In the present study, we investigated the role of miR-126 overexpression in regulating functions of normoxia- or hypoxia-injured EPCs. The underlying mechanism of PI3K/Akt/eNOS pathway was explored. Moreover, we determined whether transfusion of miR-126-primed EPCs (EPC^miR-126^) is more effective in treating ischemic stroke in mouse model. The level of cEPCs and their contained miR-126 was also examined in EPC-transfused MCAO mice.

## 2. Materials and Methods

### 2.1. Culture and Characterization of EPCs

EPCs were generated from C57BL6 mice and characterized as previously reported [19]. After 7 days of culture, the cells that double positive for Di-acLDL and Bs-Lectin staining were considered as early outgrowth EPCs.

### 2.2. EPC Transfection

The lentivirus-carrying green fluorescent protein (GFP) marker for gene expression of murine miR-126 (Lv-miR-126) or scrambled control (Lv-NC) were purchased from Genepharma (Shanghai, China). To obtain overexpressed miR-126 (EPC^miR-126^) and control EPCs (EPC^NC^), the EPCs were transfected with Lv-miR-126 or Lv-NC as previously described [20]. In brief, the EPCs were incubated with EPC culture medium containing the lentivirus for 24 h. Transduction efficiency (the expression of miR-126 in EPCs) was quantified by RT-PCR.

### 2.3. Hypoxia Model of EPCs

Hypoxia injury model of EPCs was conducted as previously described [21, 22]. In brief, EPC^miR-126^ or EPC^NC^ were subjected to hypoxia (O_2_/N_2_/CO_2_, 1/94/5) for 72 h. The cells cultured under normoxic conditions for 72 h were set as the control. Afterwards, the EPCs were used for functional analyses (proliferation, migration, and tube formation).

### 2.4. Real-Time Polymerase Chain Reaction (RT-PCR)

The level of miR-126 in the EPCs was determined using Hairpin-it™ miRs RT-PCR Quantitation kit (GenePharma, Shanghai, China) based on manufactory instruments. PCR primer are as follows: 5-TATGGTTGTTCTCGACTCCTTCAC-3 and 5-TCGTCTGTCGTACCGTGAGTAAT-3 for miR126 and 5-CTCGCT TCGGCAGCACA-3 and 5-AACGCT TCACGAATTTGCGT-3 for U6. Quantitative real-time PCR was conducted on a LightCycler 96 System (Roche Diagnostics, Switzerland). U6 was used for normalizing the data of miR-126 expression. Relative expression of miR-126 was calculated by using the 2^−ΔΔCT^ method.

### 2.5. EPC Proliferation Analysis

The proliferation ability of EPCs was measured by MTT (3-(4,5-dimethylthiazyol-2yl)-2,5-diphenyltetrazolium bromide) (5 mg/ml, Sigma) assay. The EPCs were seeded at 2 × 10^3^/well in 96-well plate with 100 *μ*L EGM-2 culture medium. MTT solution (20 *μ*L) was added and incubated with cells for 4 h at 37°C, then 150 *μ*L DMSO was added to each well and incubated with the cells for 20 min at 37°C. The optical density (OD) value of cells was read at 490 nm using a microplate reader (BioTek). Cells in triplicate wells were examined at each time point, and the experiment was repeated three times.

### 2.6. EPC Migration Analysis

The migration of EPCs was measured as previously described [23]. Briefly, a scratch was made through the cultured EPCs treated with hypoxia or normoxia (control). After 16 h cultivation, the invasion of cells into the scratched area was observed by an inverted microscope. Quantitative analysis of migration was calculated as the equation: (cell-free area at 0 h – cell-free area at 16 h)/cell free area at 0 h × 100%.

### 2.7. EPC Tube Formation Analysis

The tube formation ability was measured by using the tube formation assay kit (Chemicon) as we previously described [23]. Briefly, the EPCs were placed (1 × 10^4^ cells/well) onto the surface of the EC matrix and incubated with EPC culture medium for 12 h at 37°C. Four representative fields were taken, and the average of complete tubes formed by EPCs in the fields was counted.

### 2.8. ROS Analysis

Dihydroethidium (DHE) (Beyotime, China) staining was used to measure the intracellular ROS production as we previously described [24]. The EPCs treated with hypoxia or normoxia (control) were incubated with DHE solution (5 *μ*M) at 37°C for 2 h. Flow cytometry was used to analyze the level of ROS in EPCs. For signal pathway study, LY294002 (PI3K inhibitor; 20 *μ*M) was prior added to culture medium for 2 h.

### 2.9. NO Analysis

Total NO production of EPCs treated with hypoxia or normoxia was detected by using a Nitric Oxide Assay kit (Beyotime) as we previously described [24]. For pathway blocking experiments, the cells were preincubated with LY294002 (20 *μ*M) for 2 h.

### 2.10. Western Blot Analysis

The protein of EPCs were extracted with cell lysis buffer (Applygen Technologies Company, Beijing) supplemented with protease inhibitor tablet (Thermo Scientific). Protein lysates were electrophoresed through SDS-PAGE gels and transferred onto PVDF membranes. The membranes were blocked with 5% nonfat milk for 1 h and incubated with primary antibodies against beta actin (1 : 1000, EarthOx, San Francisco, CA, USA), PI3 kinase p110a (1 : 1000, CST, USA), Akt (1 : 5000, CST, USA), p-Akt (1 : 500, CST, USA), and p-eNOS (1 : 1000, Invitrogen, USA). Blots were developed with ECL solution (Amersham, Sweden).

## 3. Animals and Procedure

### 3.1. Animals

Adult C57BL6/J mice (6–8 weeks of age; weight ranges from 20–24 g) were purchased from the Animal Experiment Center of Guangdong Province (Guangzhou, China) and housed in the Animal Care Facility at the Guangdong Medical University. The mice were maintained in a pathogen-free environment with free access to food and water on a 12 h light/dark cycle before and after surgery. All surgery was performed under 2.5% isoflurane anesthesia, and all efforts were made to minimize pain and distress. All experimental procedures were approved by the Laboratory Animal Care and Use Committees at Guangdong Medical University.

### 3.2. Middle Cerebral Artery Occlusion Surgery (MCAO) and EPC Transfusion

The mice were included and random divided into sham, MCAO, EPC^NC^, and EPC^miR-126^ groups. Focal ischemic stroke and sham-operated mice were carried out as we reported previously [25]. Two hours after MCAO, the mice (*n* = 10/group) were administrated via the tail vein with phosphate-buffered saline (PBS, vehicle), EPC^NC^, or EPC^miR-126^ (2 × 10^6^ cells/100 *μ*L in PBS). The EPCs were donated from C57BL6 mice. Three days after EPC transfusion, the brain samples of mice were used for the measurements of NDS, cerebral blood flow (CBF), infarct volume, and cerebral microvascular density (cMVD) in the peri-infarct area. The blood samples were used for the analyses of the numbers and the level of miR-126 of cEPCs. In addition, seven days after EPC transfusion, the angiogenesis in the peri-infarct area was measured.

### 3.3. Measurements of Neurological Deficit Score

On day 3, the NDS was evaluated as we previously described by using the 5-point scale method [25].

### 3.4. Measurement of CBF

The CBF of mice from various groups was measured by the PeriCam PSI System (Perimed, Sweden) as we previously described [25].

### 3.5. Measurements of Infarct Volume

On day 3 after EPC transfusion, the infarct volume of each mouse was measured by using 2% 2,3,5-triphenyltetrazolium chloride (TTC) staining as we previously described [25].

### 3.6. Immunofluorescence Analysis

cMVD in the peri-infarct area was measured by using immunofluorescence staining as described in a previous study [26]. For angiogenesis, BrdU (IP, 65 *μ*g/g per day) was administered by intraperitoneal injection after EPC infusion for 7 continuous days [19] and determined by staining with BrdU and CD31. Specifically, the brain coronal sections were incubated with BrdU (1 : 50; Abcam) and/or CD31 (1 : 50; BD Biosciences) antibody overnight at 4°C. On the second day, the brain sections were reacted with FITC-BrdU (green) and Cy3-CD31 (red) secondary antibodies (1 : 250; Invitrogen) for 30 minutes at room temperature in the dark. The positive cells in the peri-infarct area of each section were observed using a confocal microscope (Leica, TCS SP5II, Germany). Angiogenesis was determined as BrdU+CD31+ cells according to previous report [19].

### 3.7. Isolation and Analysis of cEPCs

The level of cEPCs was determined by flow cytometry as a previous study [27]. Briefly, circulating MNCs were isolated from the peripheral blood and stained with antimouse CD34-PE (AbD Serotec, Raleigh, NC) and VEGFR2-PE-Cy3 (BD, Bioscience) antibodies for 30 min at RT. Isotype-matched (IgG) nonspecific antibodies served as negative controls. After incubation, labeled EPCs were washed with PBS and resuspended with 100 *μ*L of PBS for flow cytometric analysis. The EPCs were defined as CD34+VEGFR+ cells. The number of cEPCs was described as the number of cells per microliters of whole blood.

cEPCs were further isolated by magnetic cell sorting (MACS) (Miltenyi Biotec GmbH) according to the manufacturer's instruction. The collected MNCs were incubated with 10 *μ*L of biotin-conjugated anti-CD34 antibody (Miltenyi Biotec) in a 100 *μ*L reaction volume for 10 min, followed by adding 10 *μ*L of antibiotin microbeads (Miltenyi Biotec) for 15 min. Then, the microbead-labeled MNCs were separated by using PureProteome Magnetic Stand (Millipore). After an overnight magnet separation, the microbead-bound MNCs were resuspended with 100 *μ*L PBS. The multisort release reagent (10 *μ*L; Miltenyi Biotec) was used to cleave off the microbeads. After 10 min, the cells were washed from the released fraction carefully by adding 1 mL of PBS and centrifuged at 300*g* for 10 min. The collected cells were incubated with 10 *μ*L of biotin-conjugated anti-VEGFR2 antibody (Miltenyi Biotec) in a 100 *μ*L reaction volume for 10 min, followed by adding 10 *μ*L of antibiotin microbeads (Miltenyi Biotec) for 15 min. Then, the labeled cells were proceeded to magnetic separation as mentioned above. The isolated CD34+VEGFR2+ cells were considered as cEPCs and used for miR-126 expression detection.

### 3.8. Statistical Analysis

All data were expressed as mean ± SD. Multiple comparisons were analyzed by one- or two-way ANOVA followed by Tukey post hoc tests. SPSS 23.0 statistical software was used for analyzing the data. For all measurements, a *p* < 0.05 was considered statistically significant.

## 4. Results

### 4.1. Characterization of EPCs and miR-126 Expression in EPCs

**Figure 1:**
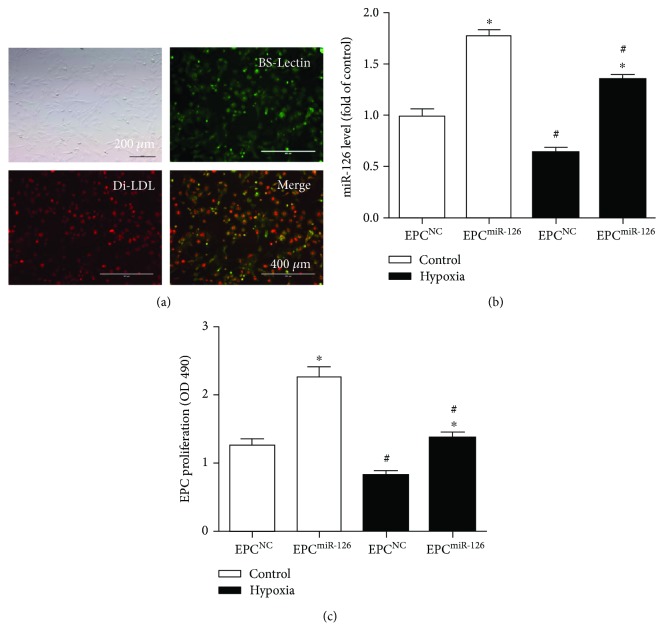
Characterization and transfection efficiency of EPCs and the effect of miR-126 overexpression on the proliferation ability of EPCs and hypoxia-injured EPCs. (a) Representative pictures showing cultured EPCs under transmitter light and fluorescent light by double-staining analysis. Red: Di-acLDL uptaking; green: Bs-Lectin staining; yellow: Di-acLDL and Bs-Lectin positive cells as EPCs. Scale bar: 400 *μ*m. (b) qRT-PCR analysis of miR-126 expression in EPCs after lentivirus-miR-126 transfection. (c) MTT assay of EPC proliferation. ^∗^*p* < 0.05 versus EPC^NC^; ^#^*p* < 0.05 versus control, *n* = 3/group.

As shown in Figure 1(a), the EPCs were defined as Di-acLDL and Bs-Lectin double-staining cells. Our qRT-PCR results showed that hypoxia significantly decreased miR-126 expression in EPCs (versus control; *p* < 0.05; Figure 1(b)). Not surprising, miR-126 transduction significantly increased the level of miR-126 in both vehicle and hypoxia-injured EPCs (versus EPC^NC^; *p* < 0.05; Figure 1(b)).

### 4.2. miR-126 Promoted the Proliferation of EPCs

MTT assay showed that hypoxia markedly decreased the proliferation of EPCs (versus control; *p* < 0.05; Figure 1(c)). Overexpression of miR-126 increased the proliferation of EPCs in both control and hypoxia groups (versus EPC^NC^; *p* < 0.05; Figure 1(c)).

### 4.3. miR-126 Promoted the Migration of EPCs

**Figure 2:**
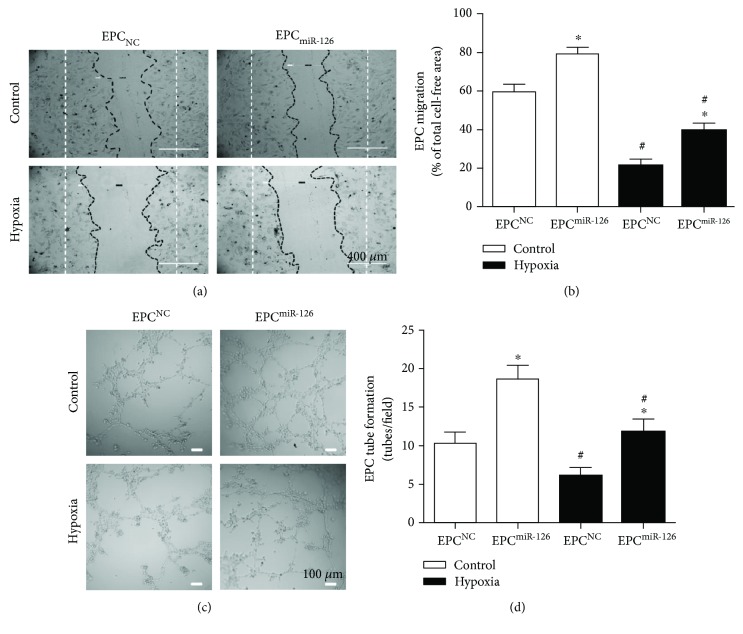
Effects of miR-126 overexpression on the migration and tube formation of EPCs and hypoxia-injured EPCs. (a) Representative image of EPC migrations. Scale bar: 400 *μ*m. (b) Summarized data on migration of EPCs. (c) Representative image of EPC tube formation. (d) Summarized data on tube formation. ^∗^*p* < 0.05*versus* EPC^NC^; ^#^*p* < 0.05*versus* control, *n* = 3/group.

As shown in Figure 2, the migration of EPCs was significantly decreased under hypoxia (versus control; *p* < 0.05; Figure 2). Overexpression of miR-126 significantly improved the migration of EPCs in control and hypoxia groups (versus EPC^NC^; *p* < 0.05; Figure 2).

### 4.4. miR-126 Increased the Tube Formation Ability of EPCs

**Figure 3:**
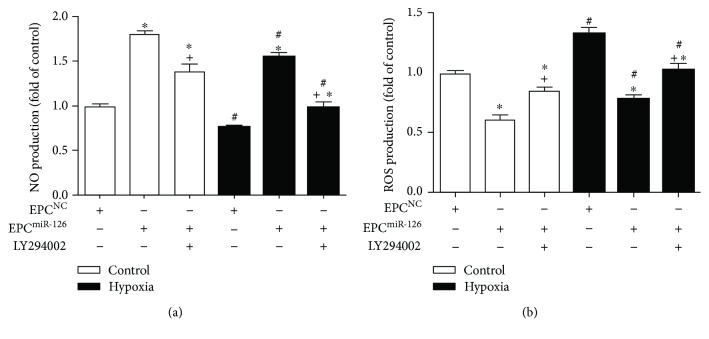
Effects of miR-126 overexpression on ROS and NO production in EPCs and hypoxia-injured EPCs. (a) NO generation of EPCs. (b) ROS production of EPCs. ^∗^*p* < 0.05*versus* EPC^NC^; ^+^*p* < 0.05*versus* EPC^miR-126^; ^#^*p* < 0.05*versus* control, *n* = 3/group.

As shown in Figure 3, the tube formation ability of hypoxia-treated EPCs was markedly decreased (versus control; *p* < 0.05; Figure 3). After miR-126 overexpression, the tube formation ability of EPCs was significantly increased in both vehicle and hypoxia-treated groups (versus EPC^NC^; *p* < 0.05; Figure 3).

### 4.5. miR-126 Decreased ROS Production and Increased NO Production of EPCs via PI3K/Akt/eNOS Pathway

**Figure 4:**
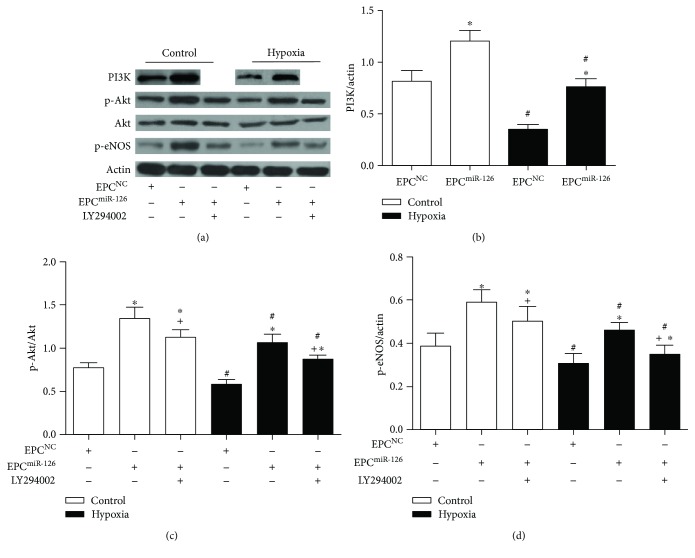
Effects of miR-126 overexpression on the expression of PI3K, p-Akt/Akt, and p-eNOS in EPCs and hypoxia-injured EPCs. (a) Western blot showing expression of PI3K, p-Akt/Akt, and p-eNOS of EPCs. (b–d) Summarized data on the expression of PI3K, p-Akt/Akt, and p-eNOS. ^∗^*p* < 0.05*versus* EPC^NC^; ^+^*p* < 0.05*versus* EPC^miR-126^; ^#^*p* < 0.05*versus* control, *n* = 3/group.

Our results showed that ROS production significantly increased while NO production markedly decreased in hypoxia-treated EPCs (versus control; *p* < 0.05; Figure 4(a)), whereas miR-126 transduction increased NO production and decreased ROS production in both control and hypoxia-treated groups (versus EPC^NC^; *p* < 0.05; Figure 4(a)). Additionally, PI3K inhibitor inhibited the beneficial effects of miR-126 on increasing NO production and decreasing ROS production of EPCs (versus EPC^miR-126^; *p* < 0.05; Figure 4(a)).

Western blot results showed that the levels of PI3K, p-Akt/Akt, and p-eNOS in hypoxia-treated EPCs were decreased (versus control; *p* < 0.05; Figure 4(b)), whereas miR-126 transduction increased the levels of PI3K, p-Akt/Akt, and p-eNOS in control or hypoxia-injured EPCs (versus EPC^NC^; *p* < 0.05; Figure 4(b)). Moreover, PI3K pathway inhibitor alleviated the effects of miR-126 on increasing p-Akt/Akt and p-eNOS expression (versus EPC^miR-126^; *p* < 0.05; Figure 4(b)). These data indicated that PI3K/Akt/eNOS pathway was implicated in the effects of miR-126 on NO production and ROS generation in EPCs.

### 4.6. miR-126 Enhanced the Therapeutic Effects of EPCs on Decreasing Cerebral Injury and Improving Neurological Deficits and CBF in MCAO Mice

**Figure 5:**
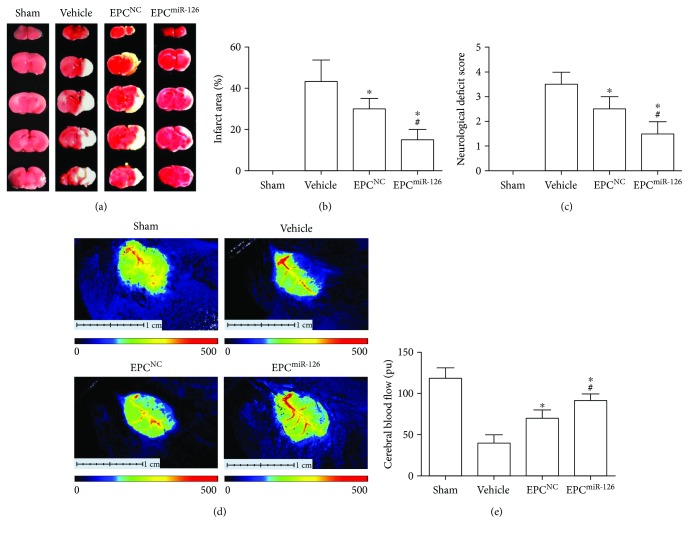
miR-126 priming enhances the efficacy of EPCs in decreasing infarct area and NDS and improving CBF in MCAO mice. (a) Representative images of TTC staining. (b) Quantitative analysis of infarct size in different groups. (c) NDS in different groups. (d) The representative images of CBF. Blue to red represents low to high perfusion. (e) Summarized data on CBF. ^∗^*p* < 0.05*versus* vehicle; ^#^*p* < 0.05*versus* EPC^NC^, *n* = 10/group.

As shown in Figure 5, the EPCs significantly reduced infarct volume and NDS (versus vehicle, *p* < 0.05; Figures 5(a)–5(c)). Meanwhile, the EPCs also improved the CBF (versus vehicle; *p* < 0.01; Figures 5(d), 5(e)). What's more, miR-126 overexpression significantly promoted the therapeutic effects of EPCs on increasing CBF, reducing infarct volume and NDS of MCAO mice (versus EPC^NC^; *p* < 0.05).

### 4.7. Infusion of miR-126-Primed EPCs Enhanced the Efficacy in Increasing cMVD and Promoted Angiogenesis in the Peri-Infarct Area of Ischemic Damage

**Figure 6:**
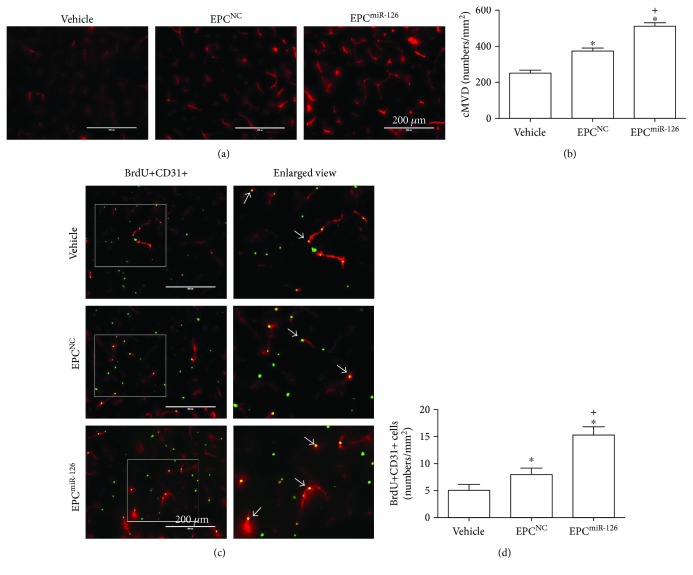
miR-126 priming enhances the efficacy of EPCs in increasing cMVD and angiogenesis in the peri-infarct area of MCAO mice. (a) Representative pictures of cMVD (CD31 immunostaining). Scale bar: 200 *μ*m. (b) Summarized data of cMVD. (c) Representative pictures of angiogenesis (BrdU+CD31+ cells). Red, CD31 for vessel; green, BrdU for new generated cells; pink, double staining. Scale bar, 200 *μ*m. The enlarged view of double-staining cells is in the left panel of each image. The arrows indicate the double staining cells. (d) Histogram showing the number of BrdU+CD31+ cells. ^∗^*p* < 0.05*versus* vehicle; ^+^*p* < 0.05*versus* EPC^NC^, *n* = 10/group.

EPCs increased the cMVD in the peri-infarct area in MCAO mice (versus vehicle; *p* < 0.05; Figure 6). As expected, the transfusion of miR-126-primed EPCs enhanced the efficacy (versus EPC^NC^; *p* < 0.05; Figure 6). Moreover, as shown in Figure 6, EPC transfusion increased angiogenesis (versus vehicle; *p* < 0.05; Figure 6). The transfusion of miR-126-primed EPCs showed better efficacy in promoting angiogenesis (versus EPC^NC^; *p* < 0.05; Figure 6).

### 4.8. miR-126-Primed EPCs Increased the Level of cEPCs and Their Contained miR-126

**Figure 7:**
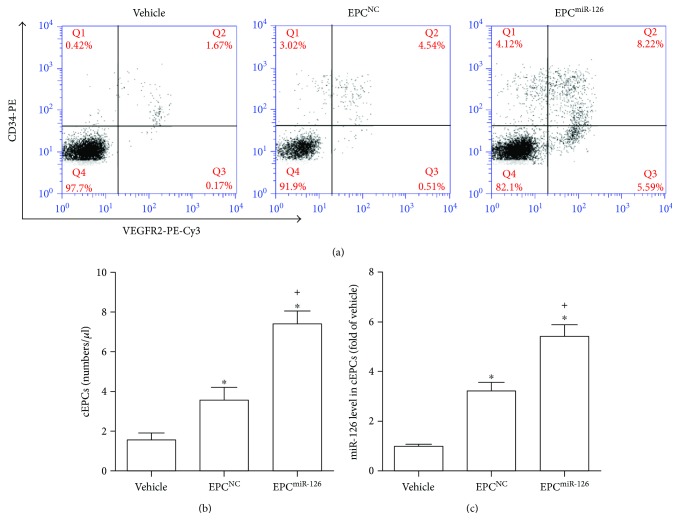
miR-126 priming enhances the efficacy of EPCs in increasing cEPCs and the level of miR-126 of cEPCs in MCAO mice. (a) Representative flow cytometry plot of the levels of cEPCs in each group. (b) Summarized data on the levels of cEPCs. (c) The miR-126 level of cEPCs in MCAO mice in each therapeutic group. ^∗^*p* < 0.05*versus* vehicle; ^+^*p* < 0.05*versus* EPC^NC^. cEPCs: circulating EPCs, *n* = 10/group.

As shown in Figure 7, the infusion of EPCs increased the levels of cEPCs after 3-day transfusion (versus vehicle; *p* < 0.05; Figure 7(a)). Infusion of miR-126-primed EPCs further increased the level of cEPCs at this time point (versus vehicle; *p* < 0.05; Figure 7(a)). Furthermore, the infusion of EPCs increased miR-126 expression in cEPCs (versus vehicle; *p* < 0.05; Figure 7(b)). In addition, the infusion of miR-126-primed EPCs was more effective to increase miR-126 expression in cEPCs (versus EPC^NC^; *p* < 0.05; Figure 7(b)).

## 5. Discussion

Ischemia- and hypoxia-induced energy failure, loss of cellular ion homeostasis, free radical and cytokine-mediated toxicities, inflammation, and disruption of the blood-brain barrier are major pathophysiological processes of ischemic stroke [28]. Current treatments for ischemic stroke mainly rely on vascular recanalization including intravenous or intra-arterial fibrinolysis and interventional treatments [29–31]. However, due to the complicated pathophysiological processes, approaches for promoting cerebral recovery following ischemic stroke are limited. Emerging studies demonstrate the effects of EPCs in accelerating cerebral recovery after ischemic stroke [32]. Upon cerebral ischemia, the EPCs are mobilized from the BM to injured blood vessels and ischemic brain tissue. The recruited EPCs then differentiated into mature ECs and secrete protective cellular factors, which participate in endothelialization and replacement of damaged ECs in ischemic tissue [33]. What's more, the EPCs participated in promoting neovascularization by abundant growth factors and cytokines, such as VEGF, SDF, and IGF-1, which can promote EC viability and tube formation, reduce cell apoptosis, and recruit endogenous progenitor cells [34]. Taken together, these findings suggest that EPCs are closely related to the vascular homeostasis and remodeling after ischemic stroke and highlight the potential of EPCs as a cell candidate for regenerative therapy.

However, the *in vivo* pathological environment may influence the cell fate and therapeutic benefit of EPCs [7, 8, 27]. Reports have showed that the clonogenic and adhesion ability of EPCs was reduced in diabetic patients with peripheral artery disease [35]. Our recent study demonstrated that the migration and tube formation abilities of EPCs were decreased in human renin and angiotensinogen transgenic mice [19]. Another research also demonstrated that the levels of cEPCs were decreased in the plasma of the diabetic mice following ischemic damage [27]. These evidences indicated that the functions of EPCs were closely related to vascular diseases. As a critical factor in cerebral ischemia, hypoxia could impair functions of EPCs [21, 36]. Notably, shorter-term hypoxia showed better proliferation, enhancements in tube-like structure formation, and motility of EPCs, as well as mRNA expression of VEGF, CXCR4, PI3K, and Akt [37, 38], while these effects were reversed by prolonged hypoxia [22], which consisted with our present study demonstrated that long-time hypoxia induced EPC dysfunction (decreased proliferation, migration, and tube formation abilities). Thus, in order to enhance the therapeutic effect of EPCs in cerebral ischemia injury, gene transfection and pretreatment have been used to modify EPCs. Various genes such as CXCR4 [39], ACE2 [19], VEGF [40], and eNOS [41] have been transduced into EPCs, which play a beneficial role in improving the therapeutic efficacy of EPCs.

In the present study, we transfected EPCs with miR-126, which is highly enriched in ECs and plays a pivotal role in maintaining vascular integrity and promoting angiogenesis [42]. In vascular injury, the endothelial levels of miR-126 were decreased [43]. The decreased circulating miR-126 has been proved correlated to stroke [44]. Targeted deletion of miR-126 in human umbilical vein vascular endothelial cells upregulates vascular cell adhesion molecule-1, which in turn promotes plaque formation by enhancing leukocyte adherence to the endothelium [45]. The level of miR-126 in EPCs from diabetes mellitus (DM) [18, 46], preeclampsia [47], and coronary artery disease [12] patients was reported to be abnormally lower expressed. This study, for the first time, demonstrated that the level of miR-126 was reduced in EPCs under hypoxic situation, paralleled with inhibited EPC proliferation, migration, and tube formation abilities. The proliferation, migration, and angiogenesis functions of EPCs play an important role in repairing vascular injury by enhancing the ability of EPCs to mobilize from the BM to circulation, migrate into injury or ischemic sites, and promote angiogenesis [48]. Additionally, miR-126 could enhance EPC homing to thrombogenic and ischemic area by targeting PIK3R2 and CXCR4, which plays an important role in the therapeutic effect of EPCs on ischemic diseases [39, 49, 50]. These data suggest that the abnormal expression of miR-126 in the EPCs might be associated with EPC dysfunction and correlated with various vascular diseases. Furthermore, we confirmed that miR-126 overexpression could promote proliferation, migration, and angiogenesis of EPCs in hypoxic or normal condition, which indicated rescuing and enhancing effects of miR-126 on EPC functions.

Hypoxia-induced oxidative stress plays critical role in cerebral ischemic injury, and the mechanisms are relevant to the reduced NO and increased ROS production [51, 52]. Oxidative stress could impair mobilization and decrease the functionality of EPCs. Evidence suggested that antioxidant treatment prevents the oxidative stress-induced EPC dysfunction [53]. As an important signaling molecule for oxidative stress, ROS plays an important role in endothelial dysfunction via altering vascular integrity [54], while NO could contribute to maintain vascular homeostasis by reducing ROS production [55]. Here, we found that miR-126 increased NO production while reduced ROS production in hypoxia-treated EPCs. These indicate that miR-126 decreases oxidative stress of EPCs, which might help improve the effect of EPCs on vascular repair. To further confirm the underlying mechanism of miR-126 involved in regulating ROS generation and NO production of EPCs, we measured the PI3K/Akt/eNOS pathway expression and performed pathway block experiments. Our result showed that miR-126 activated PI3K/Akt pathway and upregulated the downstream protein eNOS phosphorylation in EPCs, which was responsible for the production of NO. Furthermore, we found the beneficial effects of miR-126 overexpression on promoting NO generation and decreasing ROS production in EPCs were partially blocked by PI3K inhibitor. Thus, our findings provide novel evidence that miR-126 could decrease oxidative stress of hypoxia-treated EPCs via activating PI3K/Akt/eNOS signal pathway.

To explore the function of miR-126 overexpression in EPCs *in vivo*, we investigated the therapeutic efficacy of EPC^miR-126^ on MCAO mice model. Our data showed that transfusion of EPC^miR-126^ enhanced the beneficial effects of EPCs in attenuating cerebral damage (decreased the infarct volume and NDS) and promoting cerebral repair (increased cMVD, CBF, and angiogenesis). Angiogenesis is a pivot component of wound tissue repair processes. The EPCs are believed to play a pivotal role in angiogenesis, which represents an important endogenous tissue repair mechanism. The EPCs participate in angiogenesis by directly differentiating into matured ECs or by secreting angiogenic factors increasing the viability of resident ECs [56]. In the present study, we stained newly generated ECs with CD31 and BrdU for the index of angiogenesis [39, 57]. Our results showed that the newly generated ECs were increased in the peri-infarct area after EPC transfusion, and miR-126 promoted the efficacy of EPCs on angiogenesis. Moreover, new vessel formation after stroke has been shown to replenish blood flow to the ischemic area of the brain [58]. Reestablishing cerebral blood flow in ischemic cerebral microvascular bed contributed to the restoration of neurovascular function and has been set as a potential treatment strategy for ischemic stroke [59]. In this study, we demonstrated for the first time that miR-126 promoted the efficacy of EPCs on ameliorating CBF of ischemia-injured mice, which indicated the beneficial effects of EPC^miR-126^ on angiogenesis may contribute to the CBF preservation and subsequently neurological functional improvement. Our findings demonstrated that EPC transplantation decreased NDS of ischemic mice, which is consistent with a recent study demonstrating that EPC transplantation improved long-term neurobehavioral outcomes of ischemic stroke [60]. More importantly, we found that miR-126 overexpression could enhance this effect of EPCs. As been proved that angiogenesis contributes to the therapeutic effect of EPCs in promoting neurogenesis after ischemic stroke [32], the beneficial effect of EPC^miR-126^ on angiogenesis may also contribute to neuronal functional recovery after ischemic stroke. Taken together, all these data indicate the advantages of miR-126 in enhancing therapeutic efficacy of EPCs for ischemic stroke.

Additionally, we measured the cEPC numbers and the level of miR-126 in cEPCs after transfusion. Accumulating evidence demonstrates that the level of cEPCs is reduced in various stroke risk factors such as hypertension [61], diabetes [62], and atherosclerosis [63]. The number of cEPCs has been recently reported to positively correlate with reendothelialization ability after vascular injury [64]. Thus, we also compared the level of cEPCs between different groups. As a result, we found the level of cEPCs was decreased in MCAO mice. Furthermore, we found that infusion of EPC^miR-126^ increased the level of cEPCs and their contained miR-126. As the level of cEPCs negatively correlates with severity of ischemic damage and independently associated with outcome of ischemic stroke [65], the increasing numbers of cEPCs and miR-126 level in cEPCs after transfusion of EPC^miR-126^ may be an underlying mechanism involved in the therapeutic efficacy for ischemic stroke.

## 6. Conclusion

Our findings showed that miR-126 rescued/enhanced multiple functions of EPCs, decreased oxidative stress of EPCs via regulating PI3K/Akt/eNOS pathway, and improved EPC-based therapeutic effects on ischemic stroke. Moreover, circulating EPCs and their carried miR-126 might be important factors closely related to the therapeutic effect of EPCs for ischemic stroke. Our data provide a strong rationale for using miR-126 target to develop vasoprotective drugs and enhance the efficacy of EPC-based therapy.
